# Identification and functional analysis of NAD^+^ metabolism-related gene NT5E in pulmonary hypertension

**DOI:** 10.3389/fgene.2026.1787122

**Published:** 2026-04-07

**Authors:** Yanan Chu, Jinxiu Yang, Tianxin Ye, Fangcong Yu, Qian Wang, Jingming Zhuo

**Affiliations:** 1 Department of Geriatric Medicine, Shandong Provincial Hospital Affiliated to Shandong First Medical University, Shandong First Medical University, Jinan, Shandong, China; 2 Department of Cardiology, the First Affiliated Hospital, School of Medicine, Zhejiang University, Hangzhou, Zhejiang, China

**Keywords:** GEO, machine learning, NAD+, NT5E, pulmonary hypertension, WGCNA

## Abstract

**Background:**

Pulmonary hypertension (PH) is a severe progressive disease characterised by elevated pulmonary vascular resistance and right ventricular hypertrophy. Increasing evidence has highlighted the vital role of nicotinamide adenine dinucleotide (NAD^+^) metabolism in cardiovascular disease. However, the role of NAD^+^ metabolism-related genes (NMRGs) in PH remains unclear. In this study, we aimed to identify novel NMRGs as biomarkers in PH.

**Methods:**

Using the Gene Expression Omnibus database and Limma R package, we identified differentially expressed genes (DEGs) of PH and downloaded NMRGs from Kyoto Encyclopedia of Genes and Genomes and Reactome databases. Candidate NMRGs were subsequently identified by overlapping DEGs, NMRGs, and module genes obtained by weighted gene co-expression network analysis. The diagnostic value of these candidate NMRGs was evaluated using receiver operator characteristic (ROC) curve analysis, and gene set enrichment analysis (GSEA) was performed to explore the functional roles of hub genes. CIBERSORT algorithm was employed to assess immune cell infiltration in the PH microenvironment. Finally, the functional role of target genes in PH was validated through *in vitro* cellular experiments.

**Results:**

Through comprehensive bioinformatics analyses across multiple datasets, we identified two NMRGs: NT5E and CD38. ROC analysis confirmed the higher predictive accuracy of NT5E, with area under the ROC curve values reaching 0.891 and 0.894 in GSE113439 and GSE53408 datasets, respectively. GSEA revealed that patients with high NT5E expression exhibited significant enrichment of PH-related biological functions and pathways. Given the relationship between NAD^+^ and immunity, immune infiltration analysis was performed, which showed a close association between NT5E expression and plasma cells and eosinophils in the PH microenvironment. *In vitro* experiments further showed that NT5E was significantly upregulated in a PH cell model, and knockdown of NT5E attenuated hypoxia-induced proliferation, resistance to apoptosis, and migratory ability of pulmonary arterial smooth muscle cells.

**Conclusion:**

Based on bioinformatics analysis and *in vitro* validation, we confirmed that NMRGs affect PH progression, with NT5E showing potential as a novel diagnostic marker and therapeutic target in PH.

## 1 Introduction

Pulmonary hypertension (PH) is a group of disorders characterised by advanced pulmonary artery remodelling, which leads to high pulmonary vasculature resistance, right ventricular hypertrophy, right heart failure, and death, thus, posing a serious threat to human health (Isobe et al., 2023; Humbert et al., 2019). Current advances in PH diagnosis and treatment have greatly improved the prognosis, especially with the introduction of vasodilators (Humbert et al., 2022); however, vascular remodelling changes are recalcitrant to presently available treatments, and lung transplantation remains the ultimate therapy in severe cases (Masson et al., 2022). To provide better options for early diagnosis and treatment of PH, exploring the signature genes that are closely related to the development of PH is crucial.

Nicotinamide adenine dinucleotide (NAD^+^) functions as an essential coenzyme in redox reaction, immune modulation, and DNA damage repair, playing a vital role in various biological processes including metabolic functions and proliferative process in cancer, metabolic disorders, and cardiovascular and pulmonary diseases (Kane and Sinclair, 2018). A balance between NAD^+^ biosynthesis and breakdown is vital to maintain cellular NAD^+^ metabolism homeostasis (Zapata-Pérez et al., 2021). NAD^+^ metabolism homeostasis and NAD^+^ biosynthesis pathways influence gene amplification and epigenetic remodelling, which are closely related to the progression of various cancers (Chelvanambi et al., 2020). Importantly, PH is a cardiopulmonary disease with cancer-like characteristics. For example, many studies have linked nicotinamide phosphoribosyl transferase amplification and vascular remodelling in PH (Chen et al., 2017). The NAD^+^-dependent protein, SIRT6, participates in the regulation of hypoxia-induced imbalance of proliferation and apoptosis of human pulmonary arterial smooth muscle cells (PASMCs). Targeting *SIRT6* and the related downstream metabolism signalling pathways may be a novel strategy for the treatment of hypoxia-induced pulmonary artery hypertension (Li et al., 2023). Recently, the homeostatic function of NAD^+^ was shown to be pivotal in the pathogenesis of PH, and increasing NAD^+^ level could ameliorate PH symptoms in animal models (Zhang et al., 2024). Therefore, treatment with NAD^+^ precursor may reverse PH phenotypes, providing a new therapeutic strategy for PH treatment. However, the specific mechanism of NAD^+^ metabolism on PH remains unknown, and research on combined PH and NAD^+^ metabolism-related genes (NMRGs) is limited. Therefore, investigating NMRGs in PH may lead to new therapeutic strategies that can alleviate vascular remodelling, providing new directions for the research and treatment of PH. This integrated approach has the potential to uncover novel biomarkers, therapeutic targets, and personalised treatment strategies for patients with PH. Therefore, in-depth exploration in this field is required to broaden our understanding of PH and bridge the gap between basic research and clinical applications, ultimately improving patient outcomes.

To explore possible pathogenesis, we used the Gene Expression Omnibus (GEO) database to analyse differentially expressed genes (DEGs) between control and PH samples. The weighted gene co-expression network analysis (WGCNA) R package was then used to find suitable module genes. The DEGs were merged with key module genes and NMRGs for intersection to identify differentially expressed NMRGs (DE-NMRGs). In addition, two machine learning algorithms and two validation datasets were applied to identify target genes. Gene set enrichment analysis (GSEA) and receiver operator characteristic (ROC) curve analysis were used to further validate selected target genes and examine the level of immune cell infiltration associated with the expression of these genes. Finally, the target gene NT5E which with higher diagnostic efficiency was selected for cellular experiments to validate its relationship with PH progression. Our findings may provide new insight into the role of NMRGs in the prognosis and diagnosis of PH.

## 2 Materials and methods

### 2.1 Data acquisition

We downloaded transcriptome data from PH related microarrays (GSE113439, GSE53408, and GSE117261) from the GEO database (https://www.ncbi.nlm.nih.gov/geo/). To identify the gene expression profiles for PH, we used the GSE113439 dataset, which included 11 controls and 15 patients with PH. For validation, we used the GSE53408 and GSE117261 datasets, which comprised 12 patients with pulmonary arterial hypertension and 11 controls and 58 patients with pulmonary arterial hypertension and 23 controls, respectively.

NMRGs were acquired from the Kyoto Encyclopedia of Genes and Genomes (KEGG) (hsa00760) and Reactome (R-HSA-196807) databases. After removing duplicates, 51 NMRGs were identified (Supplementary Material 1).

### 2.2 Identification and enrichment analysis of DEGs between PH and controls

DEGs between PH and control samples in the GSE113439 were identified using R package ‘Limma’, with adjusted *p*-value less than 0.05 and |log_2_^fold-change^| greater than 0.5; they were visualised as volcano plot and heatmap using the ‘ggplot2’ package. Biological functional enrichment analysis of these DEGs was conducted by Gene Ontology (GO) and KEGG pathway enrichment analyses using the ‘org.Hs.eg.db’ package. GO analysis included the biological process (BP), cellular component (CC), and molecular function (MF) categories. The cutoff criterion was defined as a *p*-value less than 0.05.

### 2.3 WGCNA

The ‘WGCNA’ package in R software was employed for WGCNA analysis of GSE113439, examining the correlation between modules and PH (Langfelder and Horvath, 2008). Specifically, clinical traits were classified into two types, control and PH, to identify module–trait relationships and the correlation matrix. The appropriate soft-thresholding power was chosen with an ideal soft thresholding power of 5. For each expression profile, module membership and gene significance were defined as the correlation values for each trait and module eigengene. The most relevant module to PH was identified as a key module for subsequent analysis.

Finally, the obtained DEGs, 51 NMRGs, and genes in the key module were intersected, and the intersected genes were described as DE-NMRGs and filtered by machine learning.

### 2.4 Screening target genes by machine learning

Two machine learning models were used, Least Absolute Shrinkage and Selection Operator (LASSO) logistic regression and Random Forest (RF), to screen out target genes from the DE-NMRGs using the ‘glmnet’ and ‘randomForest’ packages in R, respectively. LASSO logistic regression analysis is a data mining method that compresses estimation method to obtain a more refined model by constructing a penalty function (Friedman et al., 2010). RF analysis is a decision tree-based machine learning method that focuses on evaluating the significance of variables by scoring the importance of each variable (Alakwaa et al., 2018). In the present study, the predictive genes were obtained separately by LASSO regression and RF algorithm, and the intersection of the two machine learning algorithms was identified as target genes.

### 2.5 ROC curve analysis

Alignment diagram of biomarkers were constructed using the rms package in R (Sachs, 2017). The predictive power of the alignment diagram was assessed using calibration and decision curves. Multivariate modelling with combined selected genes was used to identify biomarkers with high sensitivity and specificity for PH diagnosis by using a visualisation tool (https://hiplot.com.cn/basic/roc). The ROC curves were plotted and area under the ROC curve (AUC) was calculated separately to evaluate the performance of each model using the R packages ‘pROC’ (Robin et al., 2011). AUC > 0.8 indicated that the model had a good fit.

### 2.6 GSEA

We performed a single-gene GSEA to further investigate the potential mechanisms and pathways of target genes. The following thresholds of significance were considered: absolute NES values >1, adjusted *p*-values <0.05, and FDR q-values = 0.25.

### 2.7 Immune cell infiltration estimation

To quantify immune cell infiltration using transcriptome data, the ‘CIBERSORT’ R package (CIBERSORT R script v1.03; http://cibersort.stanford.edu/), based on a deconvolution algorithm, was used. Immune cell infiltration between PH and normal samples was compared using the Wilcoxon test, and the results were visualised by violin diagrams drawn using the ‘ggplot’ package.

### 2.8 Rat PASMC culture and treatment

Rat PASMCs were purchased from Pricella Life Science & Technology (Wuhan, China) and cultured in Dulbecco’s modified Eagle medium (Beyotime Biotechnology, China) supplemented with 20% foetal bovine serum (Gibco, New Zealand). The cells were incubated at 37 °C in 5% CO_2_. The cells were cultured under normoxia (containing 21% O_2_) or hypoxia (containing 1% O_2_) condition for 24 h. Cells between passages 3 and 5 were used for further experiments. At least 3× were repeated, respectively.

To knockdown NT5E expression, PASMCs were transfected with small interfering RNA (siRNA) targeting NT5E (Tsingke Biotech, Beijing, China). According to the instructions of lipofectamine 3000 transfection reagent, primary rat PASMCs were transiently transfected with si-NT5E or si-NC for 24 h, exposed to hypoxia (1% O_2_) or normoxia condition for another 24 h and used in the subsequent experiments. The following siRNAs against NT5E were used:

si-NT5E #1: 5′- GGU​UGU​GAA​UGU​AAG​CGA​A -3′

si-NT5E #2: 5′-GGU​UGA​GUU​UGA​UGA​UAA​A-3′

si-NT5E #3: 5′-CAG​UUG​AAG​GUC​GGA​UCA​ATT-3′

si-NC: 5′-UUC​UCC​GAA​CGU​GUC​ACG​U-3′

### 2.9 RNA extraction and quantitative reverse-transcription polymerase chain reaction (qRT-PCR)

Total RNAs were isolated from rat PASMCs using RNA isolater Total RNA Extraction Reagent (Vazyme, China), according to the manufacturer’s instructions. Total RNAs were converted into cDNA using a reverse transcription reagent kit. The cDNA was amplified with gene-specific primers using ChamQ SYBR qPCR Master Mix (Vazyme, China) in a 20 μL reaction volume. Rat β-actin was used as an internal control. Relative RNA levels were calculated using the 2^−△△CT^ method. The following primers were used: NT5E, 5′-CAA​GTG​TCG​AGT​GCC​CAT​CTA​TG-3′ (Rat-NT5E-Forward); and β-actin, 5′-AGA​TCA​AGA​TCA​TTG​CTC​CTC​CT-3′ (Rat-β-actin-Forward).

### 2.10 Western blot

PASMCs were harvested and lysed in the RIPA lysis buffer (Servicebio, China) containing a complete protease inhibitor cocktail for 15 min on ice to extract the total protein. Extracts were centrifuged at 4 °C for 15 min at 12,000 rpm, and the supernatants were collected and added to a loading buffer. The protein concentration was determined using a BCA protein assay kit (CWBIO, China). Lysates containing equal amounts of protein were separated using 10% SDS-PAGE and electro-transferred to a polyvinylidene difluoride membrane (BIO-RAD, United States of America). The membrane was blocked at room temperature for 2 h in 5% defatted milk in Tris-buffered saline with 0.1% Tween 20 and then incubated with primary antibodies at 4 °C overnight. Afterwards, the membranes were incubated with a horseradish peroxidase-labelled secondary antibody (1:2,000; Cell Signaling Technology) in a blocking buffer at room temperature for 2 h. The membranes were cut to blot the target and loading control proteins. An enhanced chemiluminescence reagent (Vazyme, China) was used to detect the proteins. Average protein expression was normalised to β-actin expression using the ImageJ software (http://rsbweb.nih.gov/ij/). Detailed information on the relevant antibodies is listed in Supplementary Table S1.

### 2.11 Immunofluorescence

The PASMCs were cultured and subjected to immunofluorescence staining to examine protein expression. First, the cells were plated on glass slides in 24-well plates. After treatment, the cells were fixed with 4% paraformaldehyde for 20 min and permeabilised with 0.5% Triton-X-100 for 20 min. After blocking with 5% bovine serum albumin, the cells were incubated with anti-NT5E (1:50) overnight at 4 °C. The following day, the cells were incubated with an anti-fluorescence secondary antibody (1:100) at 37 °C for 2 h. Finally, nuclear staining was performed using 4′,6-diamidino-2-phenylindole (DAPI, 1 μg/mL) for 10 min at room temperature in the dark. The cells were rinsed thrice with phosphate buffered saline prior to image acquisition using a fluorescence microscope.

### 2.12 Wound healing assay

The PASMCs were cultured in a 6-well plate after growing to 60%–80% confluence, and the monolayer was scratched using a 10 μL sterile pipette tip, with images captured at the beginning. Then, the cells were exposed to hypoxia (1% O_2_), and images were captured after 24 h using a microscope. The images were compared to quantify the migration rate of the cells.

### 2.13 Statistical analysis

Prism 10.5.0 software (GraphPad Software, Inc.) was used for statistical analysis. All data are presented as mean ± standard deviation (SD). Data among the groups were compared by two-way analysis of variance followed by Tukey’s multiple comparisons test, and data between two groups were compared using unpaired two-tailed t-test for normal distribution or Mann–Whitney U test for non-normal distribution. Difference with *p*-values <0.05 were considered statistically significant.

## 3 Results

### 3.1 DEGs screening and functional enrichment analysis

**FIGURE 1:**
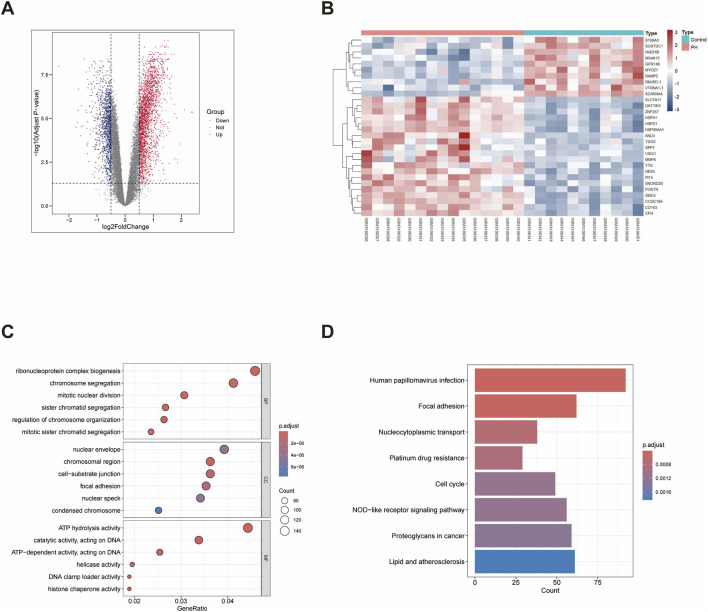
GSE113439 dataset gene differential expression analysis and enrichment analysis of DEGs. **(A)** Volcano map of DEGs between the PH and control groups in the GSE113439 dataset. **(B)** Heatmap of DEGs from the GSE113439 dataset. **(C)** GO enrichment analysis of DEGs. **(D)** KEGG pathway enrichment analysis of DEGs.

In total, 3,866 DEGs were obtained by differential expression analysis of the GSE113439 database under the criteria of adjusted *p*-value <0.05 and |log_2_^fold-change^| >0.5, with 2,559 significantly upregulated and 1,307 downregulated DEGs (Figures 1A,B). We used GO and KEGG enrichment analyses to demonstrate the biological functions and signalling pathways associated with these DEGs (Supplementary Materials 2,3). GO analysis revealed that the DEGs were significantly enriched in cellular response to ribonucleoprotein complex biogenesis, chromosome segregation, mitotic nuclear division, and other BPs (Figure 1C). In CC, the DEGs were enriched in nuclear envelope, chromosomal region, and cell-substrate junction. In MF, the DEGs were associated with ATP hydrolysis activity, catalytic activity (acting on DNA), and ATP-dependent activity (acting on DNA). KEGG analysis showed the relationship between DEGs and focal adhesion, cell cycle, and NOD-like receptor signalling pathways (Figure 1D).

### 3.2 Construction of co-expression network and identification of DE-NMRGs

**FIGURE 2:**
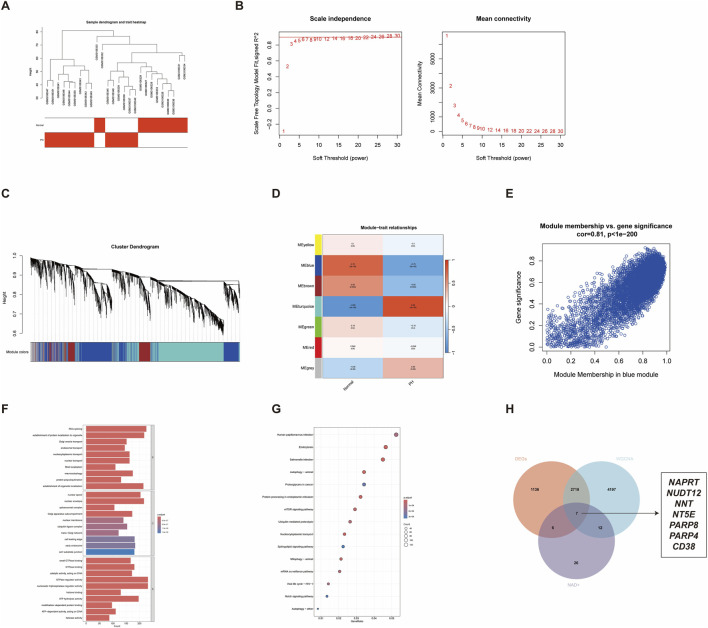
WGCNA and screening for differentially expressed NMRGs in PH. **(A)** Sample dendrogram and feature heat map were drawn based on the Euclidean distance using the average clustering method for hierarchical clustering of samples, with each branch representing a sample, height in the vertical coordinate being the clustering distance, and the horizontal coordinate being the clinical grouping information. **(B)** Optimal determination of the soft threshold for network construction. **(C)** Dendrogram illustrating gene clustering. Each leaf, a short vertical line, corresponds to a specific gene. Branches of the dendrogram group together genes that are densely interconnected and highly co-expressed. **(D)** Heatmap presenting the correlation between modules and features. **(E)** Scatterplot of significant genes versus module membership in the blue module. Gene significance (GS) and module membership (MM) correlate significantly. Pearson’s coefficient and *p*-values are indicated above each plot. **(F,G)** GO and KEGG enrichment analyses of key module genes. **(H)** Venn diagram of the intersection of DEGs, blue module genes, and NMRGs.

To further identify the central genes associated with the PH phenotype, we constructed a gene co-expression network using the WGCNA algorithm. The sample hierarchical cluster analysis results showed good clustering among the samples, with no significant outliers (Figure 2A). To construct a scale-free network, an optimal soft-threshold power of 5 was carefully selected (Figure 2B). The primed and merged modules were eventually displayed under the clustering tree (Figure 2C). Gene module identification showed the formation of seven distinct modules (Figure 2D). Given the various pathophysiological mechanisms of the PH group in the GSE113439 dataset, we choose the gene module closely linked with the recognition of control cases. Consequently, we selected the ‘blue’ module, which showed obvious proximity to normal samples (r = 0.75, *p* = 9e − 06) and significant negative correlation with PH characteristics (r = −0.75, *p* = 9e − 06) (Figure 2D). The scatter plot (Figure 2E) showed a strong correlation between GS and MM in the ‘blue’ module (Cor = 0.81, *p <* 1 × e^−200^). Furthermore, we performed functional enrichment of the ‘blue’ module genes via GO and KEGG. In the BP assessment, the genes were mostly engaged in RNA splicing, establishment of protein localisation to organelle, and other functions. These genes were localised to the nuclear speck, nuclear envelope, and other structures in CC. The gene changes associated with MF included small GTPase binding, GTPase binding and catalytic activity, and influence on DNA activity (Figure 2F). Based on KEGG analysis, these genes were particularly abundant in autophagy, endocytosis, *salmonella* infection, nucleocytoplasmic transport, protein processing in endoplasmic reticulum, and other pathways (Figure 2G). Finally, the intersection of the DEGs, WGCNA, and NMRGs was obtained using a Venn diagram; seven overlapping DE-NMRGs were selected for subsequent analysis: *NAPRT*, *NUDT12*, *NNT*, *NT5E*, *PARP8*, *PARP4*, and *CD38* (Figure 2H).

### 3.3 Identification of target genes via machine learning

**FIGURE 3:**
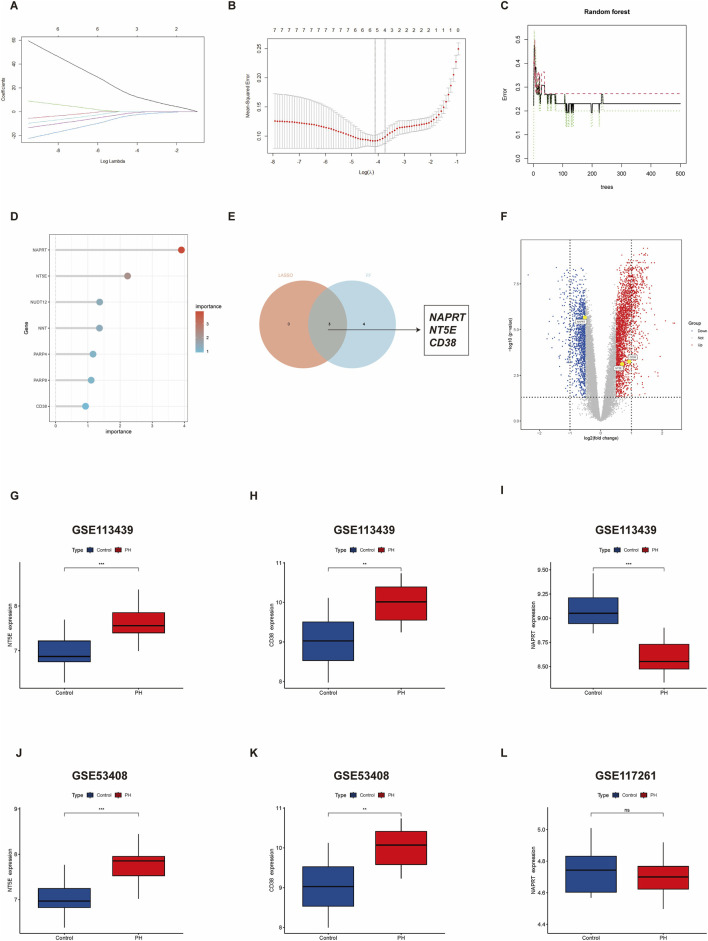
Identification of target genes via machine learning algorithm. **(A,B)** LASSO regression algorithm. **(C,D)** RF algorithm. **(E)** Venn diagrams for the two algorithms. **(F)** Volcano plot showing the three target genes in GSE113439. **(G–I)** Expression of *NT5E, CD38*, and *NAPRT* in GSE113439. **(J–L)** Expression of *NT5E*, *CD38*, and *NAPRT* in the validated datasets. PH, pulmonary hypertension; LASSO, Least Absolute Shrinkage and Selection Operator; RF, Random Forest. *, *p* < 0.05; **, *p* < 0.01; ***, *p* < 0.001; ****, *p* < 0.0001.

LASSO and RF were used to identify candidate target genes from the seven DE-NMRGs. Construction of LASSO based on 5-fold cross-validation revealed the minimal error value to successfully correspond to three characteristic genes, *NAPRT*, *NT5E*, and *CD38* (Figures 3A,B). By contrast, RF in combination with feature selection was used to determine the association between error rate and number of classification trees, and the seven significant genes by weight were selected (Figures 3C,D). After the integration of results derived from the two algorithms (Figure 3E), three target genes were obtained, *NAPRT, NT5E*, and *CD38*, shown using a volcano plot (Figure 3F).

### 3.4 Verification of the expression of the target genes in PH

Next, we validated the expression levels of the three target genes by assessing their expression in different PH-related datasets (GSE53408 and GSE117261). The expression of *NT5E* in the PH group was upregulated, compared with that in the controls (Figure 3J); similar results were obtained for the training dataset GSE113439 (Figure 3G). The expression level of *CD38* was significantly increased in the PH group compared to that in the control group in GSE53408 (Figure 3K), consistent with the results obtained from the GSE113439 dataset (Figure 3H). The expression of *NAPRT* was reduced in the PH group compared with that in the controls (Figure 3I). However, with respect to *NAPRT*, no expression was observed in GSE53408, and no significant difference in expression was observed between the PH and control groups in GSE117261 (Figure 3L). Accordingly, we further focused on *NT5E* and *CD38*.

### 3.5 Alignment diagram with good diagnostic efficacy based on target genes

**FIGURE 4:**
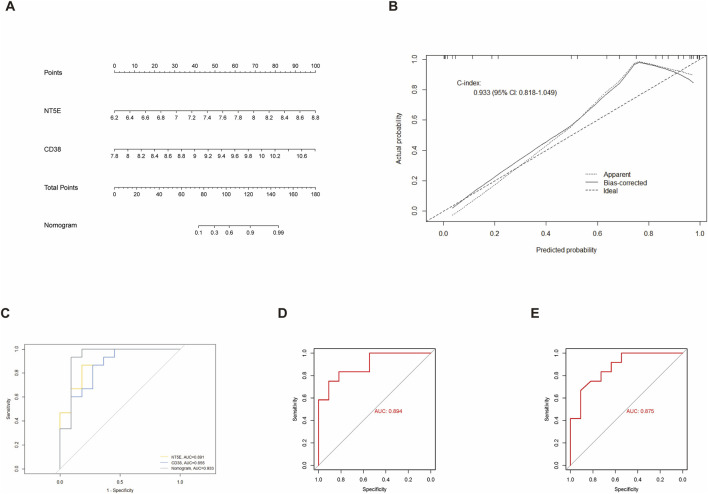
Diagnostic model construction and correlation analysis based on target genes. **(A)** Nomogram based on the expression of biomarkers *NT5E* and *CD38*. **(B)** Calibration curve of the nomogram model in **(A)**. X-axis shows the nomogram-predicted probability of PH. Y-axis shows the actual probability of PH occurrence. **(C)** AUC of *NT5E* and *CD38* in the diagnosis of PH in ROC curve multifactor analysis in the GSE113439 dataset. **(D)** AUC of the NAD^+^-related biomarker *NT5E* in the diagnosis of PH in ROC curve monofactor analysis in the GSE53408 dataset. **(E)** AUC of the NAD^+^-related biomarker *CD38* in the diagnosis of PH in ROC curve monofactor analysis in the GSE53408 dataset. PH, pulmonary hypertension; ROC, receiver operator characteristic; AUC indicates area under the ROC curve.

Based on the two target genes, an alignment diagram was constructed, and the score of each sample was calculated; the higher the total score of the patient, the higher the likelihood that the patient would develop PH (Figure 4A). The calibration curve suggested that the alignment diagram had a good diagnostic efficacy (Figure 4B). ROC univariate analysis was performed in the training and validation datasets GSE113439 and GSE53408, respectively, to assess the diagnostic value of *NT5E* and *CD38* for PH. The accuracy of *NT5E* and *CD38* in diagnosing PH in GSE113439 was 0.891 and 0.855 (Figure 4C), while it was 0.894 and 0.875 in GSE53408, respectively (Figures 4D,E). Collectively, these findings demonstrate that the NAD^+^-related biomarkers *NT5E* and *CD38* exhibit strong potential for PH diagnosis.

### 3.6 GSEA enrichment of target genes

**FIGURE 5:**
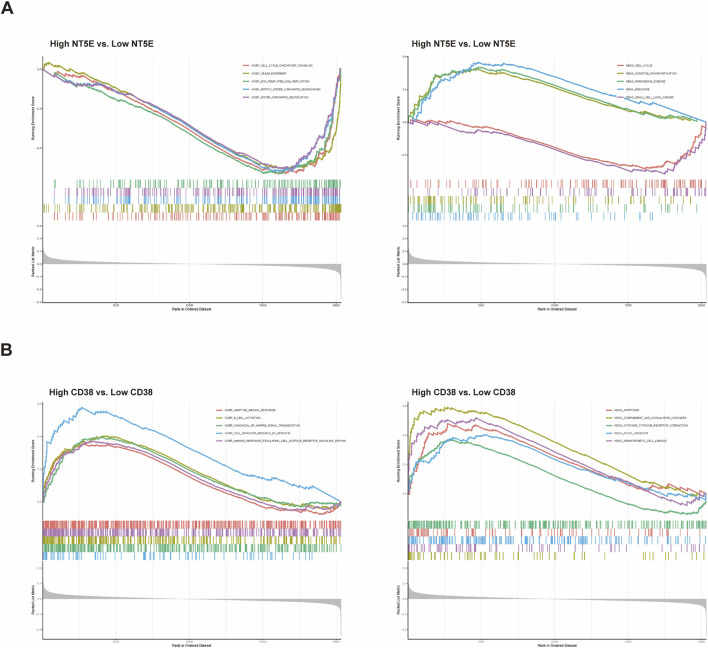
Functional and pathway enrichment in lungs from patients with PH with target genes. **(A)** Gene Ontology analysis showing biological processes enriched in the high versus low *NT5E* expression groups in the GSE113439 dataset (left). KEGG analysis showing biological pathways enriched in the high versus low *NT5E* expression groups in the GSE113439 dataset (right). **(B)** Gene Ontology analysis showing biological processes enriched in the high versus low *CD38* expression groups in the GSE113439 dataset (left). KEGG analysis showing biological pathways enriched in the high versus low *CD38* expression groups in the GSE113439 dataset (right).

According to GSEA findings (Supplementary Materials 4–7), most genes in PH samples with high *NT5E* expression were enriched in the following biological processes: cell cycle checkpoint signalling, cilium movement, mitotic sister chromatid segregation, sister chromatid segregation, and DNA replication (Figure 5A left); pathways including ribosome, Parkinsons disease, oxidative phosphorylation, small-cell lung cancer, and cell cycle were also enriched (Figure 5A right).

The *CD38* high expression group was highly enriched for cell adhesion mediated by integrin, canonical NF-κB signal transduction, B-cell activation, immune response-regulating cell surface receptor signalling pathway, and adaptive immune response (Figure 5B left); GSEA showed that complement and coagulation cascades, hematopoietic cell lineage, focal adhesion, apoptosis, and cytokine–cytokine receptor interaction were the most significantly enriched pathways in this group (Figure 5B right).

### 3.7 Immune infiltration landscape of PH and its correlation with target genes

**FIGURE 6:**
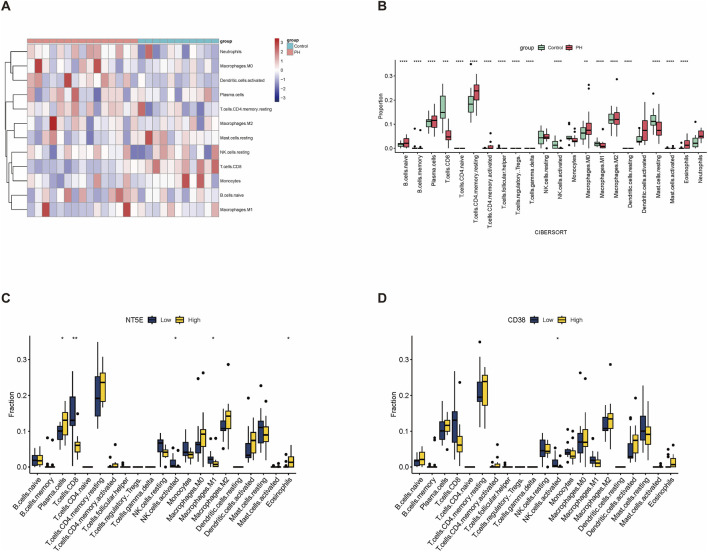
Immune infiltration landscape of PH and correlation between target genes and immunocytes. **(A)** Heatmap showing the immune cell types with the most obvious differences in PH and controls in the GSE113439 dataset. **(B)** Peripheral immune infiltration levels in the controls and patients with PH by using CIBERSORT algorithms. Violin plot showing the differences in peripheral immune infiltration score between the control and PH groups. **(C)** Peripheral infiltrating levels in the high and low *NT5E* expression groups by using CIBERSORT algorithms. Box plot showing differences in peripheral immune infiltration score between the high and low *NT5E* expression groups. **(D)** Box plot showing differences in peripheral immune infiltration score between the high and low *CD38* expression groups. *, *p* < 0.05; **, *p* < 0.01.

Considering the relationship between NAD^+^ and immunity, we used the CIBERSORT algorithm to further investigate the differences in immune cell infiltration between the PH and control groups (Supplementary Material 8). We first compared the proportion of 28 immune cell infiltration in PH and controls. Sixteen differently expressed immunocytes were identified in the PH group, including B cells, T cells, natural killer (NK) cells, monocytes, macrophages, mast cells, and eosinophils (Figures 6A,B). Subsequently, we investigated the differences in immune cell infiltration between target genes. We first compared the proportion of 22 infiltrated immune cells in the high and low *NT5E* expression groups. Plasma cells and eosinophils were significantly upregulated in the high *NT5E* expression group (Figure 6C). Further, compared with that in the low *CD38* expression group, only the infiltration of activated NK cells was lower in high *CD38* expression group (Figure 6D).

### 3.8 Increased NT5E expression in experiment PH cell model

**FIGURE 7:**
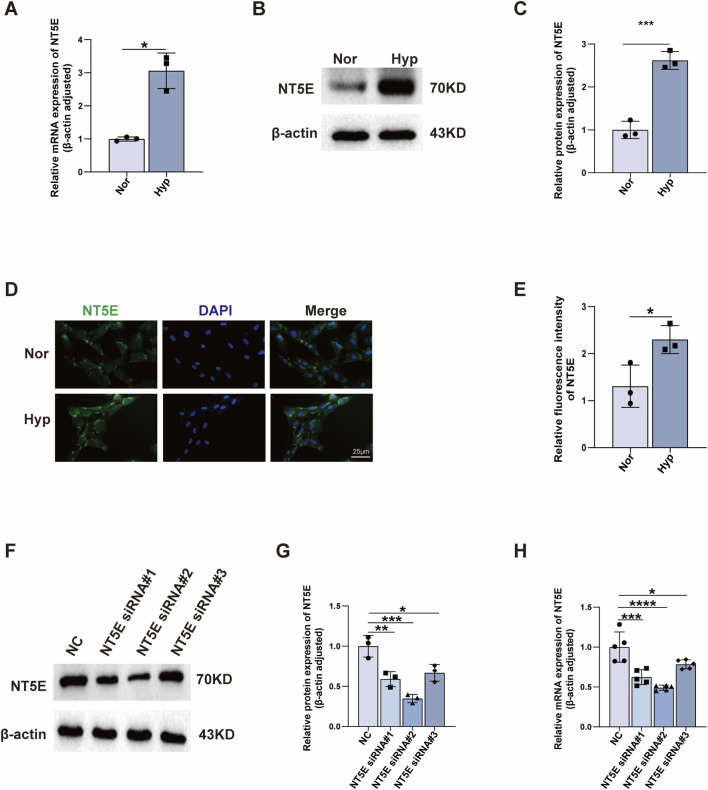
Validation of *NT5E* in a PH cell model. **(A)** Increased mRNA expression of NT5E in hypoxia-induced PASMCs. qRT-PCR analysis of NT5E mRNA in PASMCs under normoxia (21% O_2_) or hypoxia (1% O_2_) for 24 h (n = 3). **(B,C)** Increased protein expression of NT5E in hypoxia induced PASMCs. Western blot analysis of NT5E expression in PASMCs under normoxia (21% O_2_) or hypoxia (1% O_2_) for 24 h (n = 3) with β-actin as the loading control. **(D,E)** Protein content of NT5E in PASMCs was stained with anti-NT5E (*green*) and DAPI (*blue*) by immunofluorescence **(D)**; quantitative analysis showing increased expression of NT5E in PASMCs (E, *n* = 3). **(F–H)** Silencing effect of NT5E siRNA determined by western blot (F and G, n = 3) and qRT-PCR (H, n = 4). PASMC, pulmonary arterial smooth muscle cell; DAPI, 4′,6-diamidino-2-phenylindole; Nor, normoxia; Hyp, hypoxia 24 h. Data are shown as mean ± SD. *, *p* < 0.05; **, *p* < 0.01; ***, *p* < 0.001; ****, *p* < 0.0001.

To identify pivotal regulators of PH development, we focused on *NT5E*, given its higher diagnostic capacity in PH. We first analysed *NT5E* expression in the PH cell model. We further performed qRT-PCR and western blot experiments. To stimulate potential microenvironmental encountered within the pulmonary vascular bed of PH, PASMCs were subjected to hypoxia (1% O_2_) for 24 h. During the process of hypoxia treatment, qRT-PCR and western blot results showed that the mRNA and protein levels of NT5E were significantly increased after 24 h of hypoxia in PH cell models (Figures 7A–C and Supplementary Material 9). To further validate the expression of NT5E in PASMCs, immunofluorescence was performed and revealed that the protein level of NT5E was overexpressed in hypoxic PASMCs (Figures 7D,E), consistent with the RNA-seq results. Taken together, these results strongly suggest the abundance of NT5E in PASMCs upon hypoxia stimulation. Based on the expression level, NT5E was knocked down in PASMCs for the subsequent assays. NT5E siRNA was transfected into PASMCs (Figures 7F–H), and we choose NT5E siRNA#2 for further studies.

### 3.9 NT5E deficiency suppresses proliferation and promoted apoptosis in PASMCs

**FIGURE 8:**
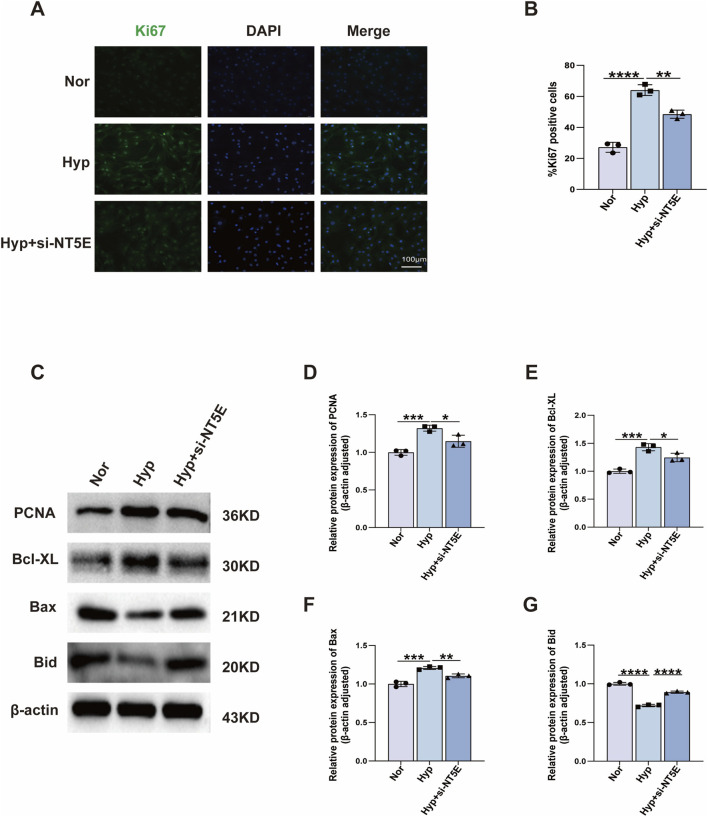
NT5E deficiency suppresses proliferation and promoted apoptosis in PASMCs. **(A)** Representative expression of Ki67 (*green*) detected by immunofluorescence staining, DAPI was used to label the nucleus (*blue*), and the merged images of Ki67 and DAPI are shown on the right. *NT5E* knockdown inhibits the expression of Ki67. **(B)** Bar graph showing the ratio of Ki67-positive cells (n = 3). **(C)** PASMCs were transfected with si-NT5E for 24 h, then exposed to hypoxia (1% O_2_) or normoxia for 24 h. Western blot analysis of protein levels PCNA, Bcl-XL, Bax, Bid, and β-actin. **(D–G)** Bar graph showing the statistic results of protein levels (n = 3). PCNA, proliferating cell nuclear antigen; DAPI, 4′,6-diamidino-2-phenylindole; PASMC, pulmonary arterial smooth muscle cell; Nor, normoxia; Hyp, hypoxia 24 h. Data are shown as mean ± SD. *, *p* < 0.05; **, *p* < 0.01; ***, *p* < 0.001; ****, *p* < 0.0001.

PH is characterised by increased PASMCs proliferation and reduced apoptosis. To examine the regulatory role of NT5E in PASMCs function, we cultured PASMCs and knocked down NT5E via siRNA. The expression of Ki67 was detected by immunofluorescence staining. Ki67 was upregulated by hypoxia treatment, and *NT5E* deficiency decreased the expression of Ki67 (Figures 8A,B). We also verified proliferating cell nuclear antigen and Bcl-XL expression in PASMCs by western blot assay and obtained similar results (Figures 8C–E and Supplementary Material 9). By contrast, PASMCs apoptosis was increased by NT5E knockdown, in line with the expression levels of Bax and Bid (apoptosis markers) (Figures 8C,F,G and Supplementary Material 9). Overall, these results suggest that NT5E knockdown suppressed the proliferation and enhanced the apoptosis of PASMCs under hypoxic conditions.

### 3.10 NT5E disruption attenuated the migration of PASMCs induced by hypoxia

**FIGURE 9:**
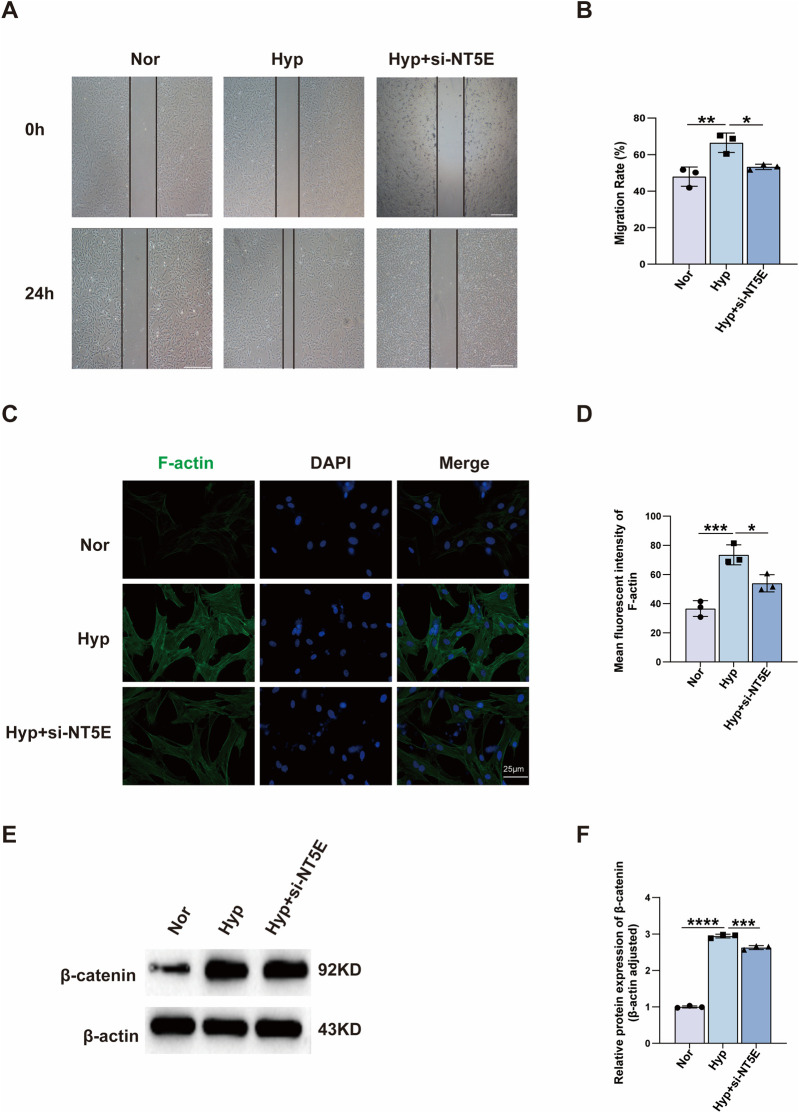
NT5E deficiency attenuates hypoxia-induced migration and reduces cytoskeleton reorganisation of PASMCs. **(A,B)** Wound healing experiments show that NT5E knockdown inhibits hypoxia-induced PASMCs migration; wounds were pictured after hypoxia exposure for 0 and 24 h (n = 3). **(C)** Rhodamine-phalloidin staining for cytoskeleton F-actin (*red*); DAPI was used to label the nucleus (*blue*). **(D)** Quantitative analysis of immunofluorescence showing the decreased F-actin expression in si-NT5E PASMCs (n = 3). **(E,F)** Western blot analysis of β-catenin protein expression of PASMCs after hypoxia treatment for 24 h (n = 3). PASMC, pulmonary arterial smooth muscle cell; Nor, normoxia; Hyp, hypoxia 24 h. Data are shown as mean ± SD. *, *p* < 0.05; **, *p* < 0.01; ***, *p* < 0.001; ****, *p* < 0.0001.

Hypoxia-induced PASMC migration is a key element of vascular remodelling during the development of PH. We examined the effect of NT5E on hypoxia-induced migration and cytoskeleton reorganisation of PASMCs. First, through wound healing assay, we found that knockdown of NT5E effectively inhibited hypoxia-induced migration of PASMCs (Figures 9A,B). Second, rhodamine-phalloidin staining for F-actin revealed that NT5E deficiency reduced cytoskeleton reorganisation of PASMCs (Figures 9C,D). To further explore the underlying mechanisms, β-catenin protein was detected by western blot. NT5E deletion decreased β-catenin protein level (Figures 9E,F and Supplementary Material 9).

## 4 Discussion

PH, which includes a heterogenous group of disorders with increased pulmonary arterial pressure, is a life-limiting pulmonary vascular disease characterised by pulmonary vasoconstriction and vascular remodeling (Hemnes et al., 2022). The complex molecular mechanisms of pulmonary arteries occlusive remodelling, which includes excessive proliferation of pulmonary artery vascular cells, extracellular matrix deposition, and perivascular inflammatory cell and cytokines infiltration, pose challenges for the treatment of PH (Yan et al., 2020; Christou and Khalil, 2022). Despite the substantial progress made in the management of PH over a long period in the past, the disease remains life threatening (Ghofr et al., 2025). Therefore, identification of novel therapeutic targets and the development of a personalised medicine approach are important. NAD^+^ is a redox cofactor and substrate that influences various cellular processes including metabolism, genomic stability, cell viability, inflammation and aging (Covarrubias et al., 2021; Verdin, 2015; Katsyuba et al., 2020). Enhanced NAD activity through addition of precursors possibly increased the activity of NAD-related enzymes and potentially improved tissue functions (Katsyuba et al., 2020). Prolonged disruption of NAD^+^ metabolism impairs multiple physiological functions involved in serious conditions such as non-alcoholic fatty liver disease (Aggarwal et al., 2023), diabetes (Berthiaume et al., 2019; Chandrasekaran et al., 2022), cardiovascular diseases (Lin et al., 2021), neurodegenerative disease (Hou et al., 2021), aging (Covarrubias et al., 2021), and malignant diseases (Navas and Carnero, 2021), highlighting the importance of NAD^+^ homeostasis in organismal health. Accumulating evidence has suggested that the above-mentioned processes, such as inflammation and redox reaction, play vital roles in the pathogenesis of PH (Hennigs et al., 2021; Geng et al., 2025). Besides, PH is often likened to malignant diseases and shares numerous pathogenic mechanisms with tumours, including hyperproliferation, resistance to apoptosis, and metabolic transitions (Pullamsetti et al., 2017). However, the mechanism by which NAD^+^ occurs in PH remains unclear. With the advancement of microarray and sequencing technologies, the exploration of the potential mechanism and diagnostic and prognostic biomarkers of PH has become convenient. The present study used bioinformatics methods to identify the key genes and pathways linking NMRGs to the pathogenesis of PH, aiming to provide a new foundation for the treatment of PH.

We used the GEO database to investigate the gene expression levels of controls and PH groups; 3,866 DEGs, including 2,559 upregulated and 1,307 downregulated genes, were identified. Subsequent GO enrichment analysis showed all DEGs were mainly associated with ribonucleoprotein complex biogenesis, components of the nuclear envelope, and ATP hydrolysis activity, while KEGG enrichment analysis showed some correlation with focal adhesion and lipid and atherosclerosis, along with NOD-like receptor signalling pathway. For a better understanding of the role of NAD^+^-related genes in PH progression, candidate models correlated with PH were identified using WGCNA analysis; DEGs and NAD^+^-related genes were further intersected, thereby improving the identification of key genes. Two critical machine algorithms found three NAD^+^-related target genes—*NAPRT, NT5E*, and *CD38*. Of them, only *NT5E* and *CD38* exhibited consistent expression trends across different datasets. We analysed the correlation between target genes and PH group by ROC analyses; the results showed that *NT5E* had higher diagnostic efficacy, compared with *CD38*. Accordingly, we choose *NT5E* as the target gene for further investigation. *NT5E* expression level was upregulated in PASMCs in the PH model. Of note, *NT5E* expression had strong correlations with PASMCs over-proliferation, apoptosis resistance, and migration induced by hypoxia. These results highlight *NT5E* as a potential diagnostic marker and treatment target for PH.

NT5E, a surface enzyme, converts extracellular adenosine monophosphate into anti-inflammatory and immunosuppressive adenosine (Gao et al., 2014). Bulk RNA-sequencing data revealed high *NT5E* expression in severe tumour specimens, which is closely related to cancer progression (Koivisto et al., 2019; Yu et al., 2021; Wang et al., 2012; Xia et al., 2023). PH and cancer have several crucial similarities and molecular features, such as altered crosstalk between cells from different tissue types, proliferative and pro-survival phenotypes, glycolytic metabolic switch, and the involvement of the immune system (Antigny et al., 2025). Therefore, the similarities between PH and cancers indicate *NT5E* as a potential valuable target for personalised treatment in PH. Our investigation revealed that in PH cell model, both mRNA and protein levels of NT5E were significantly upregulated in the PH group compared with control, implying a role for *NT5E* in PH progression. Knockdown of NT5E could significantly inhibit the over-proliferation, apoptosis resistance, and cell migration—induced by hypoxia. Notably, GSEA indicated that NT5E plays a crucial role in regulation biological processes of cell cycle checkpoint signalling, which may be involved in PASMC proliferation. Therefore, it is reasonable to suspect that NT5E may play a role in the development of PH, but elucidation of its specific mechanism would need more in-depth research. Besides, addressing unresolved issues about the role of NT5E in PH will bridge tumour and cardiovascular research, thereby offering hope for the currently incurable disease.

CD38, is a multifunctional type II and III cell surface glycoprotein involved in numerous physiological and pathological conditions, including aging, neurodegenerative diseases, and tumorigenesis (Guo et al., 2025). CD38 was found to mediate diverse activities, including signal transduction, cell adhesion, cyclic ADP-ribose synthesis, immune response, and cell proliferation (Liao et al., 2017). Various studies have indicated that CD38 overexpression enhances tumour cell proliferation and inhibits apoptosis (Wang et al., 2025). Cellular behaviours of PH are similar to those of tumour cells. In our study, we found an aberrantly increased expression of the NAD hydrolase CD38 in PASMCs. Reportedly, CD38 impacts pathways that control cell proliferation, including Notch signalling, E2F targets, and G_2_-M checkpoint (Chmielewski et al., 2018). CD38 protein possesses ADP-ribosyl cyclase and cyclic ADP-ribose (cADPR) hydrolase activities and mediates cADPR synthesis and degradation (Dogan et al., 2024). These two metabolites of CD38 catalysis play a key role as second messengers in cellular Ca^2+^ mobilisation and are closely related to elevated levels of free intracellular calcium (Li et al., 2018; B et al., 2024). The dysregulation of intracellular calcium homeostasis has been considered key mechanisms underlying the pulmonary vascular remodelling in PH (Balistrieri et al., 2023). Abnormal increase in intracellular free calcium concentration leads to excessive proliferation, migration, and contraction of the PASMCs, resulting in pulmonary vasoconstriction and remodeling (Liao et al., 2021). However, the exact molecular mechanisms and functional significance of related regulatory networks require further experimental validation.

NAPRT is a rate-limiting enzyme that catalyses the first step of the Preiss–Handler NAD^+^ biosynthesis pathway and has emerged as a promising antitumor target (Duarte-Pereira et al., 2021; Ghanem et al., 2024). NAPRT silencing leads to NAD^+^ metabolome depression, which is important for metabolic conversion of cancer cells (Navas and Carnero, 2021; Moreira et al., 2016). Low NAPRT expression correlates with poor prognosis in several human cancer type (Wu et al., 2026). In addition to cancer, the role of NAPRT in inflammatory diseases, such as obesity, type 2 diabetes, metabolic syndromes, and atherogenic inflammatory diseases, was recently discovered and is brought about by the extracellular form of NAPRT (Managò et al., 2019; Audrito et al., 2020). Inflammation is involved in all types of PH, and infiltration of perivascular inflammation aggravates pulmonary vascular remodeling (De Bie et al., 2025; Su et al., 2025). Therefore, reduced NAPRT expression in PH may enhance the activation of inflammatory pathways and increase tissue damage. However, there was no significant difference in the expression of *NAPRT* between PH and control groups in the validation datasets, possibly due to the limited sample size. The relationship between NAPRT and the occurrence and progression of PH requires further investigation.

The infiltration of immune cells, such as B cells, T cells, macrophages, and mast cells in the pulmonary arteries, is a key factor in promoting the development of PH (Ferrian et al., 2024; Li et al., 2024). For example, immune cells such as macrophages and T cells exhibit significant infiltration in PH and drive vascular remodelling through smooth muscle cell modulation, suggesting that immune infiltration is a key target for the treatment of PH (Ferrian et al., 2024). Furthermore, vascular inflammatory conditions were associated with the regulation of T cell and NK cell functions. Importantly, vascular inflammation plays vital roles in pulmonary vascular modification (Stacher et al., 2012). CD38 is widely expressed in many immune cells such as lymphocytes, dendritic cells, macrophages, monocytes, and NK cells, and is closely related to immune response (Glaría and Valledor, 2020). A majority of immune infiltration research has been carried out in PH lung tissue, with fewer studies involving CD38 expression-related immune cell infiltration in PH. Hence, we evaluated the types of immune infiltration cells in high CD38 expression group, aiming to explore the potential mechanisms of immune infiltration in PH. The proportions of B cells, CD4^+^ T cells, and macrophages were higher in high CD38 expression group than in low CD38 expression group. In keeping with the results of immune cell infiltration, GSEA analysis of groups with different CD38 expressions revealed that B cell activation, immune response regulation cell surface receptor signalling pathway, and adaptive immune response were enriched in high CD38 group. However, how the expression of the target genes influences the immune response in PH warrants further study.

This study has some limitations. First, the sample size of patients used in this study was small; larger sample size is required validate the stability of the research results. Second, we validated the NAD^+^ metabolism-related biomarkers only through *in vitro* experiments; further *in vivo* experiments should be conducted to verify the specific mechanisms and therapeutic potential of the biomarkers screened. Finally, although we identified a link between target genes and immune infiltration in patients with PH, the interpretation of immune profiles between NAD^+^-related genes and PH remains largely unexplored; larger, more diverse studies are need to confirm and build on our findings.

## 5 Conclusion

In this study, we provide novel insight into NT5E regulation and highlight potential therapeutic strategies targeting NT5E for PH treatment. NT5E loss-of-function inhibits the hyperproliferation, apoptosis resistance, and migration in PASMCs, thereby serving as a new treatment target for PH. Further research will be conducted to explore the precise molecular mechanism and functional pathway of NT5E in PH through *in vivo* experiments.

## Data Availability

The original contributions presented in the study are included in the article/Supplementary Material, further inquiries can be directed to the corresponding authors.
