# Supplementary material for: Identification and functional analysis of NAD+ metabolism-related gene NT5E in pulmonary hypertension
Source: Front Genet. 2026 Apr 7;17:1787122. doi: 10.3389/fgene.2026.1787122 (PMC13095183; doi:10.3389/fgene.2026.1787122)
Supplement: Supplementary file 2 [file Table1.docx]

**Supplementary Table S1.**

Detailed information on relevant antibodies.

| **Antibody name** | **Commercial source** | **Catalog number** | **Application** |
| --- | --- | --- | --- |
| NT5E | Abclonal | A25914 | WB and IF |
| β-Catenin | Abclonal | A19657 | WB |
| PCNA | Abclonal | A12427 | WB |
| Bcl-XL | Abclonal | A23766 | WB |
| Bid | Abclonal | A0210 | WB |
| Bax | Cell Signaling Technology | 2772 | WB |
| β-actin | Proteintech | 81115-1-RR | WB |
